# Mechanisms of migraine as a chronic evolutive condition

**DOI:** 10.1186/s10194-019-1066-0

**Published:** 2019-12-23

**Authors:** Anna P. Andreou, Lars Edvinsson

**Affiliations:** 1 Headache Research, Wolfson CARD, Institute of Psychiatry, Psychology and Neuroscience, King’s College London, London, UK; 2 The Headache Centre, Guy’s and St Thomas’, NHS Foundation Trust, London, UK; 3 Department of Medicine, Lund University, 22185 Lund, Sweden

## Abstract

Understanding the mechanisms of migraine remains challenging as migraine is not a static disorder, and even in its episodic form migraine remains an “evolutive” chronic condition. Considerable progress has been made in elucidating the pathophysiological mechanisms of migraine, associated genetic factors that may influence susceptibility to the disease, and functional and anatomical changes during the progression of a migraine attack or the transformation of episodic to chronic migraine. Migraine is a life span neurological disorder that follows an evolutive age-dependent change in its prevalence and even clinical presentations. As a disorder, migraine involves recurrent intense head pain and associated unpleasant symptoms. Migraine attacks evolve over different phases with specific neural mechanisms and symptoms being involved during each phase. In some patients, migraine can be transformed into a chronic form with daily or almost daily headaches. The mechanisms behind this evolutive process remain unknown, but genetic and epigenetic factors, inflammatory processes and central sensitization may play an important role.

## Introduction

Migraine is a recurrent, disabling neurological disorder, involving intense head pain and associated with other unpleasant symptoms. Migraine affects about 15% of the general population [1] and causes substantial personal suffering and impaired quality of life with a significant socioeconomic impact. The toll of chronic migraine on individual and society is even bigger, as up to 45% of patients presenting to headache clinics have daily or near-daily headaches [2, 3], with nearly half of them in need of a migraine preventive treatment [4]. The World Health Organization ranks migraine as the most prevalent, disabling, long-term neurological condition when taking into account years lost due to disability [5].

Considerable progress has been made in elucidating the pathophysiological mechanisms of migraine, associated genetic factors that may influence susceptibility to the disease and functional and anatomical changes during the progression of a migraine attack, or the transformation of episodic to chronic migraine. However, understanding disease mechanisms remains challenging as migraine is not a static disorder, and even in its episodic form migraine remains an “evolutive” chronic condition.

### Migraine as a life span disorder

Migraine is a life span disorder affecting children, adults and the elderly. The clinical presentation of migraine shows an age-dependent change with shorter duration and also occurrence of special paroxysmal symptoms like vomiting, abdominal pain or vertigo in childhood and largely an absence of autonomic signs in the elderly.

The prevalence of migraine in children varies, depending on the study and the age range of the included subjects, between 2.7% and 10.0% and in younger children (below 7 years) it does not differ between girls and boys [6, 7]. In adulthood, migraine is more prevalent in women than in men with a lifetime prevalence of 12–17% and 4–6%, respectively [8, 9]. A factor that may contribute to the increased prevalence of migraine in women compared to men in the reproductive years is estrogen withdrawal which is a reliable trigger of menstrual attacks in women [10]. Migraine in women usually declines after menopause [11, 12], indicating further the influence of hormonal changes on migraine occurrence. The prevalence of migraine in the elderly is about 3.5% with females affected ~ 2 times more often than males [13, 14].

The clinical manifestation of migraine is different in childhood from that in adulthood. Pediatric migraine is characterized by shorter attacks with the pain being less often unilateral. Accompanying symptoms include mild intolerance to light and rarely to noise [15], while vomiting and cranial autonomic features are significantly more frequent compared to adult patients [16, 17].

Beyond the influence of hormonal changes in women that can be partially responsible for the changes in the prevalence of migraine in adults and the elderly, another general feature seems to be a decrease in autonomic symptoms during aging. All these symptoms are associated with increased parasympathetic activity. A possible explanation is a change in the connectivity of hypothalamic areas to different autonomic control centres during aging in migraine [18]. Readers interested in changes in migraine symptoms during lifespan, as well as, in mechanisms that may be driving these changes, are encouraged to read a recent review by Straube and Andreou [18].

### Genetic and epigenetic component of migraine

Genetic factors may determine susceptibility to migraine, while different environmental factors can contribute to the development of a migraine attack [19, 20]. Mainly through genome-wide association studies (GWAS), which tested for differences in allele frequencies of single nucleotide polymorphisms (SNPs) over the genome in migraine patients and controls [21], it is now understood that multigenetic variants, rather than individual genes, influence the susceptibility to migraine. Although GWAS in migraine, similarly to other disorders studied with GWAS [22], failed to shed light on the molecular changes that are responsible for the evolutive nature of migraine, one can envisage that combined knowledge from many variants will highlight which molecular pathways potentially could be involved in migraine pathophysiology [20].

In the latest GWAS which included samples from nearly 60, 000 patients and over 300,000 controls, 44 SNPs were associated with migraine without aura, implicating 38 distinct genomic loci [23]. The majority of them were found to be implicated in molecular pathways related to vascular function. Other loci identified in this study, were involved in pathways related to metal ion homeostasis, leading to a rather unexpected, hypothesis that metal ion homeostasis might contribute to migraine susceptibility. Only a handful of loci was found to be involved in ion channel activity, with much less prominent signals [20]. The importance of those compared to the outcomes related to vascular function, remains a matter of debate, as this study highlighted that vascular dysfunction is of great importance in migraine susceptibility with neuronal dysfunction playing a rather secondary role [20].

Regardless of these outcomes, due to their small effect size no single SNP has any clinical use in predicting the risk of developing migraine. There is still a big challenge in the field of GWAS to link associated SNPs to actual genes and pathways. GWAS in migraine are yet to offer further knowledge on the functional consequences of the associated SNPs and how they influence susceptibility to migraine.

On the other hand, genetic studies of hemiplegic migraine, a rare monogenic forms of migraine [24] offered knowledge about specific genes that encode proteins involved in the function of ion channels and transporters. Specific mutations in these genes were studied in detailed and were shown to induce either loss or gain of function in cellular assays or in mutant murine. In brief, mutations involved in familial hemiplegic migraine were found in the genes *CACNA1A*, *ATP1A2* and *SCN1A, which* encode subunits of neuronal voltage-gated Ca_V_2.1 Ca^2+^, Na_V_1.1 Na^+^ channels, and glial Na^+^K^+^ ATPases, respectively. Interestingly a common consequence of these mutations is an increase in glutamate availability at the synaptic cleft of cells. Mutations in the *CACNA1A gene* can have as a consequence enhanced glutamate release due to enhanced calcium flux at the presynaptic terminal [25]. Mutations in the *ATP1A2* gene result in a smaller electrochemical gradient for Na^+^. One effect of this is the reduction or inactivation of astrocytic glutamate transporters, leading to a build-up of synaptic glutamate [26]. The *SCN1A* mutations can result in facilitation of high-frequency discharges that might also increase synaptic glutamate levels [27]. Thus, the neurons at glutamatergic synapses can fire at a higher frequency than they do under normal conditions and this might explain the increased susceptibility to cortical spreading depression, the underlying mechanism of migraine aura [28, 29]. Interestingly, mice carrying the *CACNA1A mutation exhibit blunted trigeminovascular nociceptive responses and calcitonin gene-related peptide (CGRP) expression* [30, 31]*.*

Although genetic factors may be involved in the evolutive processes of migraine, to date they failed to explain the pathophysiology of migraine and evolutive mechanisms. However, it is important to mention the knowledge gained from such studies, as they are part of disease mechanisms and disease susceptibility, while in the future they may be able to explain better the mechanisms that transform migraine into a chronic form in some individuals or achieve migraine freedom altogether later in life.

#### Is there a role for epigenetic mechanisms in migraine susceptibility and chronification?

Beyond genetic factors that could be responsible for migraine susceptibility and evolution to migraine chronification, epigenetic pathways through changes in DNA expression could also influence an individual’s sensitivity to migraine. Epigenetics refers to modification of gene expression without altering the underlying DNA sequence. A main epigenetic mechanism is DNA methylation, the covalent addition of a methyl group to the fifth carbon of cytosine residues, which is typically associated with gene silencing. The cause of epigenetic changes in not well understood, but it can include environmental factors, early life events, inflammation, stress and brain plasticity.

Epigenetics is a new area of research and only a handful of studies are done in migraine patients. Recently, the first genome-wide study of DNA methylation in headache chronification was published [32]. Although, several potentially implicated loci and processes were identified, only in the combined meta-analysis statistical significance was found for two CpG sites which were related to two brain-expressed genes; *SH2D5* and *NPTX2*. The *H2D5* gene encodes the SH2 domain-containing 5 protein which is thought to regulate indirectly synaptic plasticity through the control of Rac-GTP levels. The *NPTX2* gene encodes the neuronal pentraxin II protein, an inhibitor of excitatory synapses, through binding and clustering of glutamatergic AMPA receptors. Both proteins are highly expressed in the adult human brain [32].

A smaller pilot study aimed to identify changes in DNA methylation associated with headache chronification by characterising genome-wide DNA methylation levels in episodic migraineurs and patients suffering from chronic migraine with medication overuse headache (MOH), before and after detox programme. Although no statistical significance was found between the groups at different time points, some CpG sites of interest were identified, and are thought to be involved in drug addiction mechanisms and neuropsychiatric illness comorbid [33].

These preliminary data seem to support a role of epigenetic processes migraine, and theoretically they could be involved in mechanisms of brain plasticity and other migraine-specific processes. However, considering that migraine, both in its episodic and chronic form, is a complex and multidimensional disorder, all these preliminary data require replication and validation in much larger samples.

### Brain changes in the migraineur: is it a brain evolutive process?

Beyond functional changes, differences in the structural brain integrity, involving both the white and gray matter, which evolve over time, have been reported by several studies between migraine patients and controls.

The prevalence and volume of deep white matter lesions is increased in migraine patients, with women with migraine with aura having the highest prevalence [34–36]. Interestingly, these white matter lesions are not static and their development involves a gradual process for the evolution of focal invisible microstructural changes into focal migraine-related visible white matter lesions [37]. Later studies showed long-term higher incidence of deep white matter brain changes, especially among female patients. These changes were related to an increased number of new lesions rather than an increase in the size of pre-existing lesions [38]. In the ARIC MRI study [39], the authors showed that although migraine has an increased insistence of white matter lesions, there is no progression overtime. The authors suggest that the association between migraine and white matter lesions is stable in older age and may be attributable to changes occurring earlier in life [39]. Indeed, although white matter lesions are not as prevalent in children, they are not unusual [40–42]. Whether these early life changes are attributed to genetic factors, remains to be established, however, the outcomes of the GWAS on vascular factors that may contribute to migraine susceptibility, may also suggest an increased susceptibility to white matter lesions.

A number of studies showed cortical structural changes in migraine patients (detail reviews can be found here [43–47]. To this end there are conflicting results to whether there is cortical thinning or cortical thickening in the migraineur’s brain. Such changes include, increased thickening in the somatosensory cortex of patients with migraine that does not differ between patients with and without aura, decreased grey matter in cingulate cortex and reduced volume of the medial prefrontal cortex, atypical age-related cortical thinning in episodic migraine, increased thickness of the left middle frontal sulcus and the left temporo-occipital incisure, as well as, reduced thickness of the left superior frontal sulcus and the left precentral sulcus [48–52]. A more recent multi-centre 3 T MRI study utilising a large number of migraine patients [53] demonstrated significant clusters of thinner cortex in the patients with migraine compared with control subjects [54].

Gray matter changes have been reported in the region of the thalamus and a reduced striatal volume in migraine subjects with and without aura. Studies have shown broad microstructural alterations in the thalamus of migraine patients that may underlie abnormal cortical excitability. These changes involve reduced volume in thalamic nuclei with dense connections to the limbic system, including the central nuclear complex, anterior nucleus and lateral dorsal nucleus [55, 56]. Migraineurs were also found to have structural alterations of the brainstem with significant inward deformations in the ventral midbrain and pons, and outward deformations in the lateral medulla and dorsolateral pons [57].

An interesting study by Coppola and colleagues demonstrated that structural changes in the brain of episodic migraine patients without aura evolve over the course of the migraine cycle. Interictally, patients were shown to have a significantly lower gray matter density within the right inferior parietal lobule, right temporal inferior gyrus, right superior temporal gyrus, and left temporal pole when compared to healthy controls. Ictally, gray matter density increased within the left temporal pole, bilateral insula, and right lenticular nuclei, but no areas exhibited decreased density. The authors suggested that these morphometric changes between ictal and interictal phases indicate abnormal structural plasticity [58]. Whether these changes are an important mechanism of migraine pathology remains to be evaluated. If indeed these data can be reproduced, they demonstrate that evolutive processes happen in the migraineur’s brain constantly and in a cycling manner.

In CM patients, white-matter abnormalities were found in the brainstem and cerebellum [59]. Other studies showed, that CM is associated with subtle gray matter volume changes in several brain areas known to be involved in nociception/anti-nociception, multisensory integration, and analgesic dependence [60, 61]. Gray matter changes, have been reported to correlate with headache frequency assessed in both episodic and chronic migraine [60]. Recently, another study demonstrated alterations in the region of the hypothalamus, with the volume of the hypothalamus being significantly decreased in both episodic and chronic migraine patients, which in CM was positively correlated with headache frequency [62]. Bigger longitudinal volumetric neuroimaging studies with larger groups, especially on the chronification of migraine, are needed to understand the evolutive nature of these changes.

What causes these structural changes in the migraine brain is not known. Some alterations may be due to a genetic susceptibility towards developing migraine attacks. To this end, structural brain studies in paediatric migraine patients, could shed more light in the cause of this structural changes. A small MRI study showed significant alterations in brain volume. Compared to controls, paediatric migraine patients experienced a significant gray matter loss in several areas of the frontal and temporal lobes which are part of the pain-processing network, while they had increased gray matter volume of the right putamen. Between patients with aura compared to patients without aura, the left fusiform gyrus had an increased volume. In the paediatric population of migraine these structural changes were not correlated with disease duration and attack frequency [63]. A more recent study that used MRI apparent diffusion coefficient (ADC) found no volumetric changes in paediatric migraine patients, but demonstrated increased ADC in the region of the hippocampus, brain stem and the thalamus [64]. Although these studies used a smaller number of patients, data suggest that brain abnormalities do occur early on in migraine patients and the absence of correlation with patient clinical characteristics suggest that they may represent a phenotype developed as a consequence of genetic susceptibility.

Other changes could be a consequence of repeated head pain attacks. Such structural changes can be the result of brain plasticity, which is defined as the ability of the brain to modify its own structure and function following changes within the body or in the external environment. A number of CNS changes can contribute towards gray matter changes, such as synaptogenesis, angiogenesis, glia genesis, neurogenesis, increase in cell size, increase in myelin size and increase in blood flow or interstitial fluid. White matter changes are usually the result of axonal remodelling and changes in blood flow [65]. Despite the number of reports of structural changes in the migraineur’s brain, their importance in the biology of migraine remains uncertain. Nevertheless, the existence of structural changes, suggest that migraine induces progressive anatomical transformation in the brain that may have an evolutive role in disease progression and associated disability.

### The evolutive migraine attack

Migraine is cyclic disorders with a complex sequence of symptoms within every headache attack. In its episodic form, migraine is characterised by recurrent attacks involving different phases: (a). A premonitory phase prior to the onset of the actual headache, characterised by symptoms, such as excessive yawning, thirst, somnolence, food craving, cognitive difficulties, and mood changes [66] (b). Transient neurological symptoms, known as migraine aura (typically visual alterations), that occur just before the actual headache starts [67]. (c). An intense headache attack, usually involving only one site of the head, which can be exacerbated by movement and accompanied with hypersensitivity to sensory stimuli (e.g. light and smells), nausea [68]. (d) The postdrome phase which is mainly characterised by symptoms of fatigue, difficulties in concentration and comprehension, and neck stiffness [69]. During the interictal phase, although patients may appear normal, genetic predisposition and a number of triggers make them susceptible to an attack.

**Fig. 1:**
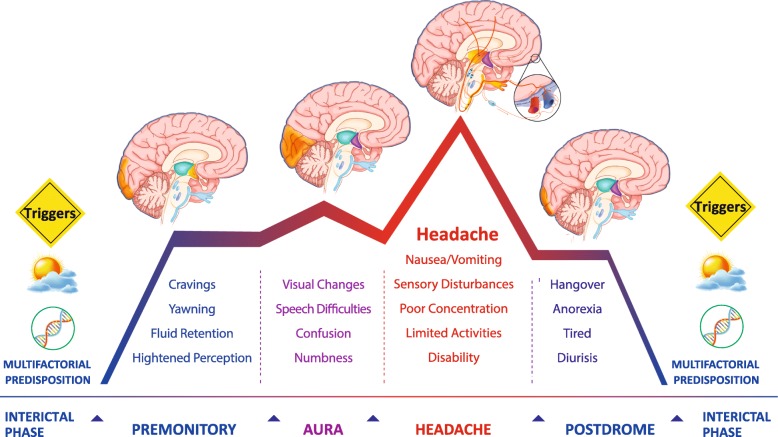
Migraine is cyclic disorders with a complex sequence of symptoms within every headache attack. In its episodic form, migraine is characterised by recurrent attacks involving different phases, with a complex sequence of symptoms within every phase. Significant advances have been made in characterising migraine as a brain disorder and in identifying evolutive functional changes in different brain areas during the different phases of a migraine attack

Several factors may trigger migraine; stress and lack of sleep are probably the most common [70]. Significant advances have been made in characterising migraine as a brain disorder and in identifying evolutive functional changes in different brain areas during the different phases of a migraine attack (Fig. 1). However, despite the number of studies on pain pathways involved during the headache phase [71], the molecular changes that actually trigger a migraine attack in the brain remain unknown. The lack of such knowledge had significantly hampered the design of migraine-specific and effective preventive treatments for a long time. Emerging evidence, partly obtained through use of the newly designed migraine treatments designed against the calcitonin gene-related peptide (CGRP) and its receptors [72], further highlights an important role of the trigeminal system in driving migraine attacks.

#### The premonitory phase and the triggering mechanisms of migraine

Accumulating evidence exists as to why the trigger of migraine attacks should be sought in the hypothalamus. The hypothalamus is a small brain structure, consisted by a number of different nuclei with distinct neuropharmacology and function. Its multitude of functions can, in a broader sense, be described as functions that organise the circadian rhythms, control and maintain the homeostasis and regulate arousal [73].

Migraine onset appears to have a circadian rhythmicity. Migraine attacks tend to occur in a daily, monthly or even seasonal pattern, further suggesting a role for hypothalamic areas, responsible for the entrained biological clock-function, in the development of the disease [74, 75]. Morphological and functional gender differences in several nuclei of the hypothalamus, may be also responsible for the monthly, menstrual associated migraine attacks, and the increased prevalence of migraine in women (~ 3:1) [76].

The premonitory symptoms of migraine are strongly associated with homeostatic functions regulated by the hypothalamus, such as arousal, sleep and feeding. The strongest, direct evidence for hypothalamic activation in migraine patients arises from brain imaging studies. These studies demonstrated, using positron emission tomography, increased blood flow in the region of the hypothalamus during the very early stages of spontaneous migraine attacks [77, 78] and during the premonitory phase of nitroglycerin (nitric oxide-NO donor)-induced migraine attacks [79].

A disturbance in homeostatic function is a significant trigger of attacks [70]. Sleep/arousal physiology in particular, deserves greater attention as sleep disturbances can trigger attacks in over 50% of migraine sufferers. Additionally, patients with both episodic and chronic migraine are more prone to have their attacks in the morning [80]. Morning headaches are also common in patients with sleep disorders, while post-operative migraine attacks in patients are common following anaesthesia [81]. Importantly, sleep itself has a striking effect as an abortive strategy [82], particularly for the majority of patients who find no relief by pharmacological treatments. The discovery of a mutation in the clock-gene *CK1δ*, causing so called familiar advanced sleep phase syndrome, was strongly linked to migraine both clinically and experimentally in mice engineered to carry this mutation [83]. The increased comorbidity of migraine in narcolepsy [84] and sleepwalking [85], also supports that migraine is an arousal-related disorder.

Such evidence highlights that the posterior region of the hypothalamus containing the circuitry for governing arousal and the transition between sleep and wake, has a key role in the triggering of migraine. However, which hypothalamic nuclei, neurotransmitters and through which mechanisms, may be implicated has not been investigated. Although several neurochemical pathways may be involved in migraine pathophysiology [86], of them, dopaminergic mechanisms appear to play some role, as yawning, a dopaminergic-driven function, is a prevalent symptom during the premonitory phase of migraine. In animal models the dopaminergic A11 nucleus of the hypothalamus has been shown to project to the trigeminocervical complex [87], an important relay system involved in migraine, and to modulate activation of the ascending trigeminothalamic pathway [88, 89]. Additionally, the A11 nucleus has been shown to be susceptible to nitric oxide donors in animal models of migraine [90, 91].

Of interest, an fMRI study of daily brain scans in a migraine patient reported a strong association for both hypothalamic and cortical activity during the premonitory phase of an attack [78]. The involvement of the occipital cortex in migraine has been long recognised, in particular because of the visual aura phenomenon. Electrophysiological studies and studies using transcranial magnetic stimulation, suggest that migraineurs have altered cortical activity, with the cortex, particularly the occipital region, appearing hyperactive [92, 93]. A study of photophobia during spontaneous migraine attacks using PET imaging also found that this migraine symptom is linked with visual cortex hyperexcitability [94, 95]. It has been suggested that thalamo-cortical dysrhythmia in migraine patients may be responsible for abnormal cortical responses [96]. Hence, a focal cortical treatment for migraine without systemic side effects is an attractive treatment opportunity. To this end, single pulse TMS (sTMS) has been shown to supress activation of the ascending trigemino-thalamic pathway [97] and is now an approved migraine treatment with good efficacy in the acute and preventive treatment of migraine [98–100].

#### The migraine aura

Migraine with aura symptoms are typically seen in about 15–20% of patients [101] and usually they develop gradually over 15–20 min and last less than 60 min [68]. It is now believed that the aura is the result of so called cortical spreading depression (CSD) [102]. fMRI studies that tried to capture or simulate aura in migraine patients also pointed to a role for CSD as a mechanism for migraine aura [103, 104]. CSD is a wave of cortical neuronal depolarisation, linked with depressed neuronal activity and blood flow changes [105], which in migraine is believed to spread out from the occipital cortex. In animals, CSD is an NMDA-receptor depended process and can be induced by cortical stimulation [106]. It remains enigmatic how CSD is triggered in patients during migraine aura. Potentially, if indeed cortex is hyperactive in patients, this hyperactivity could trigger a CSD in certain susceptible patients. As previously mentioned genetic predispositions and environmental factors may modulate individual susceptibility by lowering the CSD threshold and cortical excitation may cause sufficient elevation in extracellular K^+^ and glutamate to initiate CSD [26]. Of interest, blood flow changes suggest a functional role for the cortex and this has also been recorded in migraine patients without aura [102].

The discussion of CSD-induced headache in migraine is still a matter of debate, as not every migraine patient experiences migraine aura, while the occurrence of aura without a headache is not uncommon [107]. In experimental animal modes, CSD was shown to induce edema, reflex middle meningeal vasodilation and increases neural activity in the ipsilateral trigeminal ganglion and trigeminal nucleus [108–111]. It was demonstrated that the trigeminal activation produced by experimental CSD may cause inflammation in the meninges that occurs after the CSD has subsided [112]. Contradicting preclinical data to these previous findings also exists [113, 114]. An alternative hypothesis suggests that CSD activates cortico-thalamic fibres that in turn sensitize third order neurons of the ascending trigemino-thalamic pathway [115].

#### The headache phase

The headache phase of migraine involves activation of the ascending trigeminothalamic pathway. Through early observations in humans who underwent awake brain surgery, it became well established that the pain during a migraine attack is perceived to be felt on intracranial structures, such as, the dura matter and intracranial vasculature [116]. The sensory innervation of these structures arises from the trigeminal nerve, mainly from unmyelinated C-, and thinly myelinated Aδ-fibres, which have their cell bodies in the trigeminal ganglion. Nociceptive activation of the trigeminal fibres is referred to as “trigeminovascular activation”. The trigeminal fibres that transmit sensory information from such intracranial structures synapse on second-order neurons within the trigeminocervical complex (TCC; trigeminal nucleus caudalis, C1 and C2 spinal levels). These neurons give rise to the main ascending trigemino-thalamic pathway that relays sensory information to third order neurons, mainly in the contralateral thalamus, before processing the information to higher cortical areas.

The thalamus is a pivotal nucleus for multisensory integration and may be a strong candidate for influencing neuronal excitability in migraine. The thalamic area is a prominent site of action of triptans [117], of clinically active preventives [118, 119] and of other potential anti-migraine compounds [120]. Neuroimaging and electrophysiological studies have revealed altered network connectivity between the thalamus and pain modulating/pain encoding cortical areas during spontaneous migraine attacks, as well as, thalamo-cortical dysrhythmia which correlate with migraine symptoms [121, 122]. Recently, a dynamic functional connectivity study in migraine patients between attacks demonstrated abnormal thalamo-cortical network dynamics, with the medial and posterior thalamic nuclei identified in intrinsic subcortical connectivity networks [123]. The role of the thalamus in migraine deserves higher attention given its involvement in the development of associated symptoms, such as hypersensitivity to visual stimuli [124], while thalamo-cortical activation may also participate in the development of auditory sensitivity [125]. Furthermore, sensitization of third order thalamic neurons has been implicated in the development of non-cranial allodynia that is frequently seen in migraine patients [126].

A complex of descending networks from multiple brainstem, midbrain and cortical nuclei modulate the excitability of the ascending trigemino-thalamic pathway [127]. In the absence of any evidence of malfunction in the peripheral trigeminovascular system, a disruption of normal endogenous descending modulatory tone may play a critical role in migraine. To this end, a number of brain imaging studies showed increased blood flow in the region of the dorsal rostral pontine and brainstem in both episodic [128, 129] and chronic migraine patients [130]. A great limitation of brain imaging to date, is the lack of spatial resolution. Hence, it remains a lot of future research to delineate which descending networks and neurotransmitters that potentially are involved. Candidate nuclei include the periaqueductal gray, locus coeruleus, dorsal raphe nucleus and nucleus raphe magnus. Initially, these loci were considered as the migraine generator, due to the persistent blood flow increased in the rostral pontine area following headache relief [131]. However, the increased blood flow in this region may be expected given the role of the brainstem in descending modulatory control of pain. What is interesting is that functional connectivity fMRI studies between attacks, have identified numerous brain regions and functional networks with atypical functional connectivity in migraineurs, and demonstrated interictal impairment of the descending pain modulatory circuits, potentially indicating a reduction of pain inhibition in migraineurs [53].

**Fig. 2:**
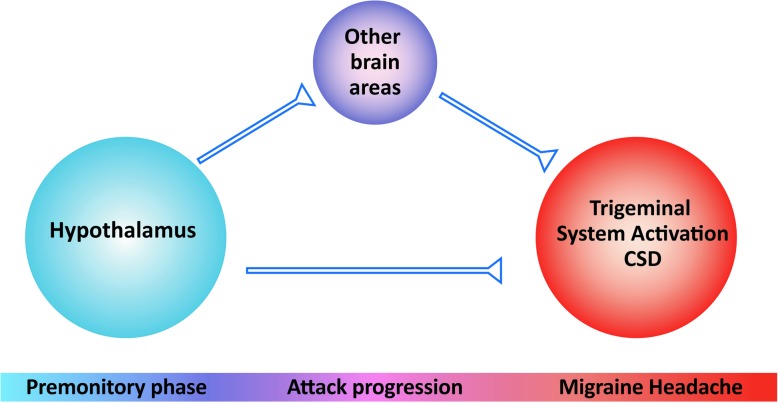
Migraine pathophysiology involves activation of the hypothalamic region during the early premonitory phase, and activation of the trigeminal system during the headache phase. Cortical spreading depression (CSD) is thought to be the biological process of the migraine aura. How activation of the hypothalamus may lead to the development of CSD and activation of the trigeminal system remains unknown. Potentially the hypothalamus may activate direct or indirect pathways involving other brain areas, such us the brainstem, or the parasympathetic system, leading to the development of migraine aura and activation of the ascending trigeminothalamic pathway

The puzzle of migraine pathophysiology is still incomplete, as we are yet to understand how hypothalamic dysfunction may lead to activation of the ascending trigeminothalamic pathway (Fig. 2). Activation of indirect pathways, involving brainstem nuclei as discussed above may indeed be a possibility. Alternatively, pathways arising from the hypothalamus that project directly to the TCC or the sensory thalamus, such as the dopaminergic A11 nucleus or the histaminergic tuberomammillary nucleus, both located in the posterior region of the hypothalamus, may directly alter the function of the ascending trigeminothalamic pathway.

An equally important pathway may be the trigeminal autonomic activation in migraine driven by the hypothalamus. Although autonomic features in migraine are not as prominent as in trigeminal autonomic cephalalgias, increased parasympathetic activity signs can be frequent [132]. Clinical evidence suggest that subjects with cranial autonomic symptoms have a hyperactive efferent arm of trigeminal autonomic reflex [132]. The hypothalamus is regulating the autonomic system and may indeed drive indirectly activation of the trigeminal system through the trigeminal-autonomic arc. The vast majority of parasympathetic fibers innervating the cerebral blood vessels originate from the sphenopalatine and the otic ganglia [133]. Sphenopalatine ganglion block in migraine patients with autonomic features was found to relieve the pain intensity by over 50% [134]. These findings suggest that increased parasympathetic tone contributes to the activation of perivascular nociceptors contributing significantly to the pain intensity and possibly to the initiation of central sensitization [135]. This could also explain a brain-driven activation of the peripheral trigeminal system and the release of CGRP from trigeminal fibers and cells located in the trigeminal ganglion. The increased cranial parasympathetic outflow and modulation of the trigeminal autonomic reflex by the hypothalamus in migraine may be of great importance.

##### The trigeminal system and its role in sustaining the head pain in migraine

Despite the various evidence of increased blood flow changes in different brain nuclei before or during the onset of the headache phase, what really alters the excitability of the ascending trigemino-thalamic pathway in a manner that a migraine headache may develop in susceptible individuals remains to be revealed. Several lines of evidence suggest that the peripheral trigeminal system is of pivotal importance in driving the headache;

- The referred pain patterns of migraine headache are similar to the locations of referred pain after stimulation of meningeal and cerebral arteries, as observed in awake patients during brain surgery [116, 136–138]. The importance of these pain-sensing structures is their vast innervation by trigeminal fibres.
- CGRP levels are increased during migraine attacks. Blood samples from patients or animal models during stimulation of the trigeminal fibres suggest that the origin of the CGRP found in migraine patients is indeed from the trigeminal nerve [139–141]. CGRP is a potent vasodilator in the periphery and a modulator of nociceptive activity centrally. On second order neurons, CGRP has no effect on spontaneous neuronal firing but it can facilitate glutamatergic activity and nociceptive activation [142–144].
- Chemicals that do not cross, the otherwise intact, blood-brain barrier (BBB) in sufferers [145–147], such as CGRP and histamine, can trigger a migraine attack [148, 149]. It is worth pointing out that the origin of the pain is not vasodilation as originally thought, as the migraine headache is not associated with cerebral or meningeal vasodilation [150], and hence anti-migraine treatments may not require vasoconstrictor properties. Additionally, healthy controls are not susceptible (or respond to a much lower degree) to migraine headache following provocation with such chemicals, suggesting that the trigeminal system in migraine patients is sensitized.
- Effective migraine treatments, like the hydrophilic sumatriptan, the large monoclonal antibodies against the CGRP system and the peripherally injected botulinum toxin [151–153], do not cross the BBB. Hence, any direct or indirect mechanism of action involves the peripheral trigeminal fibres and the trigeminal ganglion that are outside the BBB [154].

The above evidence, do not suggest that the peripheral arm of the trigeminal system is the cause of migraine, but demonstrate an important role for the peripheral trigeminal system in migraine headache. This evidence further suggests that treatments that can block activation of this system could be effective in suppressing migraine, but not necessarily the generator of migraine attacks. The trigeminal system, as well as, the trigemino-thalamic pathway in the CNS, are excitatory pathways, with glutamate being the major excitatory neurotransmitter [120]. An ideal treatment for migraine would block the glutamatergic transmission along these pathways, as this will inhibit painful signals reaching pain processing cortical centres. However, central glutamatergic blockade is challenging due to severe adverse events that can develop by inhibiting glutamatergic transmission, although not impossible [72, 118]. Peripherally acting glutamate agonists and antagonists may offer a more promising treatment approach [155, 156].

##### Vascular changes in migraine

Vascular changes in migraine were for a long time considered the driver of migraine pain. In 1940 Ray and Wolff reported that stimulation or distension of the large cranial arteries evoked head pain associated with nausea [116]. Distention of the distal internal carotid artery and middle cerebral artery during balloon inflation in patients with intracerebral arteriovenous malformations, can induce focal headache [157]. Several studies attempted to analyse the role of the dural vasodilation by measuring blood vessel diameter during an attack. A 3 T magnetic resonance angiography (MRA) study during a spontaneous migraine attack showed no significant changes of the diameter of middle meningeal artery (MMA) during a spontaneous migraine attack [158], however a larger study in cilostazol-induced migraine attacks, found that the onset of migraine is associated with increase in MMA circumference specific to the headache side [159]. In different studies in migraine patients with unilateral headache, headache was associated with intracranial dilatation of the middle cerebral artery (MCA) on the painful side, which was normalised after treatment with sumatriptan [160], as well as, with dilatation of the temporal artery [161]. Spontaneous dilatation alone cannot explain migraine pain, as arteries may dilate markedly, such as during blood pressure decreases, without induction of a migraine attack. In a nitroglycerin-triggered migraine study, peak dilation of the MCA occurred during the infusion phase of nitroglycerin. However, a migraine attack fulfilling the International Headache Society diagnostic criteria occurred ~ 5 h post infusion, suggesting a role of the cGMP pathway in the development of a migraine attack, rather than the vasodilation itself. These evidences suggest that mechanical dilatation is not adequate to activate nociceptors and cause migraine headache [162], and indeed, migraine can be induced, e.g. by sildenafil, without initial dilatation of the middle cerebral artery [163].

While it may be reasonable at this point to discard vasodilation as a direct cause of migraine, more studies are needed before eliminating blood vessels from the list of factors contributing to the pathophysiology of migraine. Both normal and pathological events occurring within and between vascular cells could mediate bi-directional communication between vessels and the nervous system, without the need for changes in vascular tone [164]. Blood vessels consist of a variety of cell types that both release and respond to numerous mediators including growth factors, cytokines, adenosine triphosphate (ATP), and nitric oxide (NO), many of which can sensitize trigeminal neurons. In addition, the majority of genomic loci identified in GWAS to be associated with migraine without aura are involved in pathways associated with vascular function [20, 23]. Hence, it could still be possible that blood vessels play a role in migraine pathophysiology in the absence of vasodilation.

#### The postdrome phase

About 80% of migraine patients report at least one non-headache symptom following the end of their headache, while the disability scores remain high [69]. The migraine postdrome is the least studied and least understood phase of migraine. Only recently, functional imaging showed widespread reduction in brain-blood flow in the postdrome, but at least some persistent blood flow increase in the occipital cortex [78, 165].

### The evolutive process of migraine chronification

Chronic migraine (CM) is a disabling, underdiagnosed and undertreated disorder, affecting ~ 1–2% of the general population [166, 167]. Progression from episodic to chronic migraine is a clinical reality [168, 169]. Studies show that each year 2.5% of episodic migraine patients progress into chronic migraine [170] which appears as a distinct entity in the classification of the International Headache Society (chronic migraine > 15 migraine days per month) [68]. The nosology of CM has several clinical implications, including the elimination of modifiable risk factors and the therapeutic preventive options for CM patients.

Patients with chronic migraine, have a significantly higher incidence of positive family history of migraine, menstrual aggravation of migraine, identifiable trigger factors, associated symptoms, and early morning awakening with headache [171]. A number of risk factors have been identified to double the risk for migraine chronification [172], including de novo increased migraine attack frequency and overuse of acute migraine medications [173–175], ineffective acute treatment that could lead to medication overuse [176], depression [177], which is a common comorbidity of migraine, and lifestyle factors such as stress, high caffeine intake and obesity [173, 178].

Certainly, either genetic factors or the presence of CM itself, induce functional and plastic changes in the brain of patients. In a recent resting-state fMRI study in chronic migraine patients without medication overuse it was shown that CM can progressively induce modifications in the CNS including large-scale reorganisation of functional cortical networks and interactive neuronal networks including the default mode network, the executive control network and the dorsal attention system [179]. These are interesting outcomes as CM can impair the attention network resulting in impairment in executive functions [179]. Additionally, a number of brain imaging studies showed changes in gray matter volume, as well as in white matter hyperintensities in CM patients, compared to episodic patients [180–184]. Whether such structural changes have any potential functional consequences remains unknow.

The physiological mechanisms that underlie the development of chronic migraine from its episodic form are not understood. Cortical excitability appears to be abnormal in chronic migraine patients, but this could be a consequence of the disease and not a driver of the chronification [185, 186]. Here we will discuss the role of inflammation and central sensitization in the evolutive process of chronic migraine.

#### Inflammation and central sensitization in the pathophysiology of migraine chronification

The question whether inflammation could contribute in the activation of the trigeminal system in a manner that could drive the migraine headache and be implicated in the evolutive process of migraine chronification remains relevant in migraine pathophysiology and treatment [187]. Indeed, the broad use of non-steroidal anti-inflammatory drugs for the acute treatment of headache supports the involvement of some neuroimmune responses in the development of migraine [188, 189], while steroid injections in the region of the greater occipital nerve, are widely used as a preventive method in chronic migraine [190, 191].

In animals, sustained CGRP release may induce peripheral sensitization [192] likely due to release of inflammatory mediators (bradykinin, prostaglandins, etc.) from nerve endings and cells of immune system [193–195]. During a migraine attack that can last for up to 72 h, the levels of the neuropeptide CGRP is increased [139]. This leads to continuous activation of C-fibers because they store CGRP and of Aδ-fibers which contains CGRP receptors. This activation may lead to production and release of inflammatory cytokines, not only in the dura, but possibly also in neuronal cell bodies, which are localized in the TG. In addition, there are CGRP receptors on ganglion cells [196]. Indeed, cytokines and chemokines may be released by neurons, microglia, astrocytes, macrophages and T cells, and activate pain neurons directly via activation of non-neuronal cells, depending on the expression of their receptors.

Major cytokines have been implicated in the pathway resulting in neurogenic inflammation, including tumor necrosis factor (TNF)-α, IL-1β and IL-6 [197]. TNF-α, a potential pain mediators in neurovascular inflammatory condition, has been suggested to be involved in the initiation and progression of a migraine attack [198]. Studies have demonstrated changes in plasma, serum, or urine levels of TNF-α in migraine patients during attacks and attack free intervals [199, 200]. Franceschini and coworkers reported that mRNA expression of TNF-α increased following migraine induction in animal models [201]. Elevated TNF-α serum levels in humans, even in outside of attacks, confirm a possible role of TNF-α in migraine [202]. A direct pathogenic role of TNF-α has not been reported during the use of this antibody in RA or MC/UC. Considering the very high prevalence of migraine, several hundred-thousand migraine patients must have received TNF-α antibody but there are no reports on any prevention of migraine attacks, not even in case-reports. This suggests that preventing TNF-α inflammation is not a viable anti-migraine target.

Although strong and direct trigeminal stimulation causes release of CGRP and substance P which may lead to neurogenic inflammation in animal models (reviewed by [198], it appears to have minor impact in acute migraine. In the line of the hypothesis that continued stimulation of both C-fibers and Aδ-fibers can cause TG inflammation and hence be implicated in chronification, this has been studied to some extent experimentally; (i) In cultured trigeminal neurons, with focus on the inflammatory pathways [203, 204]. (ii) Administered CFA (Complete Freunds’ Adjuvant) into the temporomandibular joint (TMJ), which elicited activation of trigeminal ganglion (TG) neurons [205]. (iii) Trigeminal activation using chemical stimulation of the dura mater with CFA, to test whether application of CFA on the surface of the dura mater can cause long-term activation of the TG, serving as a model of migraine chronification [206] and activation of the trigeminal nucleus caudalis leading to central sensitization [207].

The above experiments suggest that inflammation indeed could activate TG. Using culture of isolated trigeminal neurons as a model for studies of neurons and glial cells, reportedly there was enhanced expression of CGRP, and of the mitogen-activated protein (MAP) kinase both in neurons and in SGCs following inflammation. The activation of a MAP kinase–dependent inflammatory signal pathway is involved in over-expression of CGRP in nociceptive neurons and could participate in generating pain hypersensitivity [208]. Looking further into in vivo inflammation, administration of CFA into the TMJ, elicits activation of TG by increased expression of pERK1/2, pp38, CaMKII, NF-κB and DREAM after 2 and 10 days. Local inflammation in the TMJ, induced by CFA, results in an upstream inflammation response in the TG where the TMJ sensory fibers have their cell bodies. Interestingly, this involves both neurons and SGCs which together represent one anatomical and functional unit [205].

Local inflammation of dura mater can induce inflammatory activation in the TG. Application of inflammatory soup (IS) [209, 210], or CFA onto the dural surface induced changes in the expression of pERK1/2, IL-1β and CGRP positive nerve fibers in the TG illustrating that the application of inflammatory substances onto the dura mater could be used as an animal model for long term activation of the trigeminovascular system [206]. Application of CFA also induced activation (increased expression of c-Fos) of the central part of the trigemino-vascular system: the TNC and C_1_-C_2_ regions of the spinal cord [207]. Interestingly, the inflammation could be blocked by the administration of a kynurenic acid analogue (SZR72), precursor of a glutamatergic antagonist and an anti-inflammatory substance [207, 211]. All the above evidence suggest that inflammation can be responsible for the development of at least peripheral sensitization that could then lead to the development of central sensitization.

The concept of central sensitization is relevant not only for the development of chronic migraine, but also for the development of any chronic pain condition. Increased nociceptive processing, particularly due to the development of peripheral sensitization that could occur if indeed the trigeminal system is sustainably activated during migraine attacks, could lead to the development of central sensitization. Studies looking at biomarkers of functions of the trigeminal and autonomic systems identified important differences in the interictal state of chronic migraineurs compared to the interictal period of episodic migraineurs, suggesting a higher level of interictal activity of the trigeminal and cranial autonomic system in chronic migraineurs [172]. In particular, interictal levels of CGRP and vasoactive intestinal peptide (VIP) are higher in chronic than in episodic migraine [212–214]. Additionally, in animal models it has been demonstrated that chronic exposure to triptans could lead to the development of sensitization [215].

Central sensitization refers to altered behavioural of second order neurons and even of third order thalamic neurons, and is characterized by increased excitability, increase synaptic strength and enlargement of their receptive fields [216–218]. Clinically, central sensitization is manifested as a state of either hyperalgesia- an exaggerated pain in response to a stimulus that normally causes mild pain, or allodynia- a pain response to a normally non painful stimulus, and exaggerated pain response referred outside the original pain site [219]. These persistent sensory responses to noxious stimuli and long-lasting synaptic plasticity at spinal and supraspinal levels could be providing the neuronal basis for persistent pain and “pain memory” in chronic migraine [220–222]. Central sensitization is a glutamate-dependent process and at least, NMDA receptor activation seems to be pivotal for the induction of central sensitization in neuronal fibres innervating the dura matter [223].

Indeed, during a migraine headache about 80% of migraine patients develop cutaneous allodynia, characterised by increased skin sensitivity, mostly within the referred area of pain of the ipsilateral head [224, 225]. Stimulation of nociceptive afferent of the dura mater leads to a sensitization of second-order neurons receiving cervical input [226]. About two thirds of the patients developing cutaneous allodynia report that untreated migraine attacks will result in a spread of allodynia to the other side of the head or the forearm [224, 225], indicating the involvement of higher extra-trigeminal processes. The limb or upper body allodynia seen in migraineurs, and the extend of cutaneous allodynia could be due to the development and spread of neuronal sensitization from second order neurons in the TCC, to third order neurons in the thalamus [219, 224]. Hence, repeated attacks of peripheral and central sensitization could lead to the development of chronic migraine.

Central sensitization is associated with abnormal neuronal hyperexcitability in the TCC, due to an increase of the sensory inputs arriving from nociceptors on peripheral trigeminal fibres that supply the affected area, which is a consequence of peripheral sensitization [210]. Topical application of inflammatory agents on the rat dura, which induces long-lasting activation of the trigeminovascular pathway [209, 227, 228], provokes long lasting sensitization in trigeminocervical neurons that receive convergent inputs from the intracranial dura and extracranial periorbital skin. This neuronal sensitization is manifested as increased responsiveness to mechanical stimulation of the dura, to mechanical and thermal stimulation of the skin, and expansion of dura and cutaneous receptive fields [209]. These changes are parallel to an increase of the extracellular glutamate concentration of second order neurons in the TCC [229], and suggest an important contribution of glutamate and its receptors in allodynia [229].

Another factor that could contribute to the development of central sensitization and the susceptibility in developing chronic migraine could be a dysfunction in pain modulating systems. An imbalance of pain inhibition and facilitation could participate in the development or maintenance of sensitization and could contribute to the development of chronic migraine [230, 231]. What is interesting though, is that in at least 60–70% of patients, CM can be blocked by treatments that act peripherally at least on trigeminal fibres, such as the newly developed mAbs against the CGRP system and botulinum toxin. This further supports an important role of the trigeminal system as peripheral sensory inputs are important in sustaining central drive in CM.

## Conclusions

Migraine clinical and pathophysiological mechanisms are not static and evolve continuously. During lifespan the clinical phenotype of migraine changes. These changes may include transformation from episodic to chronic migraine or even a disappearance of some or all migraine symptoms all together. Genetic and epigenetic susceptibility may be responsible for such changes, although to date, studies failed to shed any light on how such genetic alterations may be responsible for migraine pathophysiology or any evolutive mechanism. On the other hand, anatomical changes in the brain of a migraine patient exist even from early childhood, but they do not seem to have any functional consequences. The causality dilemma of whether such changes are responsible for how migraine evolves, or whether migraine mechanisms drive these anatomic changes, remains to be answered. Even in its episodic form, migraine is an evolutive condition with different mechanisms involved in the evolutive process of a migraine attack. These mechanisms include hypothalamic alterations during the premonitory phase, cortical excitability in the aura phase, activation of the ascending trigeminothalamic pain pathway with an involvement of the peripheral trigeminal arm during the headache phase, and potential cortical changes during the postdrome phase. How migraine headache is triggered following hypothalamic activation remains unknown. A potential involvement of the parasympathetic pathway is possible, as it could be influenced by hypothalamic changes and in turn activate the trigeminal system through the trigemino-autonomic arc. Such a mechanisms could explain activation of the peripheral trigeminal system from a brain-initiated event. The mechanisms that underlie the development of chronic migraine from its episodic form are not well understood. Several factors have been identified to increase the risk for migraine chronification. Inflammation and central sensitization play a significant role in the evolutive mechanisms of chronic migraine.

The continuous changes in migraine phenotype and pathophysiology during a migraine attack between episodic and chronic migraine and during the patient lifespan, make migraine, even in its episodic form, a chronic evolutive disease.

## Data Availability

N/A.
